# Immunogenic Domains and Secondary Structure of *Escherichia coli* Recombinant Secreted Protein *Escherichia coli*-Secreted Protein B

**DOI:** 10.3389/fimmu.2017.00477

**Published:** 2017-04-24

**Authors:** Bruna Alves Caetano, Letícia Barboza Rocha, Eneas Carvalho, Roxane Maria Fontes Piazza, Daniela Luz

**Affiliations:** ^1^Laboratório de Bacteriologia, Instituto Butantan, São Paulo, São Paulo, Brazil; ^2^Centro de Biotecnologia, Instituto Butantan, São Paulo, São Paulo, Brazil

**Keywords:** *Escherichia coli*-secreted protein B, *Escherichia coli*, protein structure, peptide sequence, immunogenic domain

## Abstract

Several pathogenic bacteria are able to induce the attaching and effacing (A/E) lesion. The A/E lesion is caused by effector proteins, such as *Escherichia coli*-secreted protein B (EspB), responsible together with *Escherichia coli*-secreted protein D for forming a pore structure on the host cell, which allows the translocation of effector proteins. Different variants of this protein can be found in *E. coli* strains, and during natural infection or when this protein is injected, this leads to variant-specific production of antibodies, which may not be able to recognize other variants of this bacterial protein. Herein, we describe the production of a hybrid recombinant EspB toxin that comprises all known variants of this protein. This recombinant protein could be useful as an antigen for the production of antibodies with broad-range detection of EspB-bearing bacteria, or as an antigen that could be used in vaccine formulation to generate antibodies against different EspB variants, thereby increasing immunization potential. In addition, the recombinant protein allowed us to analyze its secondary structure, to propose the immunogenic regions of EspB variants, and also to characterize anti-EspB antibodies. Our results suggest that this hybrid protein or a protein composed of the conserved immunogenic regions could be used for a variety of clinical applications.

## Introduction

Gram-negative pathogenic bacteria, such as enteropathogenic *Escherichia coli* (EPEC), enterohemorrhagic *Escherichia coli* (EHEC), and *Citrobacter rodentium* are able to induce attaching and effacing (A/E) lesion (1–3). The A/E lesion is characterized by intimate intestinal epithelium adhesion, microvillus effacing, pedestal formation for effector protein translocation and the aggregation of actin and other cytoskeletal elements at the bacterial binding sites, caused by effector proteins, which are secreted into the enterocyte by a type III secretion system (T3SS) (4). The genes encoding the T3SS are located in pathogenicity islands and have many conserved structural components. The system structure consists of a syringe-like conformation, with a protein complex anchored on the bacterial membrane and a needle-shaped protein crossing the extracellular space to the host membrane, where a pore for the translocation of effector proteins is assembled (5, 6).

Enteropathogenic *Escherichia coli* and EHEC are the main bacterial agents associated with diarrhea among children under 5 years old, and both pathogens are able to induce the A/E lesion (7). Among the virulence factors comprising the T3SS of these bacteria are the secreted proteins (Esps). The Esp responsible for the syringe-like structure of T3SS is secreted protein A (EspA), which is the needle-shaped protein of approximately 25 kDa, while secreted proteins B [*Escherichia coli*-secreted protein B (EspB)] and D [*Escherichia coli*-secreted protein D (EspD)] are responsible for the pore structure assembled in the eukaryotic membrane (8).

*Escherichia coli*-secreted protein B is approximately 37 kDa in size and forms the pore assembled “needle tip” in the host cell membrane together with EspD. Also, EspB participates in phagocytosis evasion and binding to eukaryotic cell myosin, inhibition of actin interaction, and damage to the microvilli (9). There are three variants of EspB, i.e., α, β, and γ, where the α variant is subdivided into 1, 2, and 3. Allele frequency studies have shown α EspB to be the most prevalent, followed by β EspB (5, 10–13). The EspB genetic sequence varies between all variants, as demonstrated by the necessity of different primer sets for DNA amplification in gene detection studies. However, there is no clear correlation between an EspB protein subtype and a specific serogroup of EPEC and EHEC (11–13).

Several studies have used EspB protein as an antigen for the recognition of EPEC and EHEC strains (14–17), but they employed an EspB obtained by *espB* gene amplification from specific EPEC strains—mainly the prototype (E2348/69; O127:H6). Thus, the antibodies generated are against the specific EspB variant present in these strains. Therefore, the detection coverage in these methods is limited by the variant strain, which may result in other variants not being effectively recognized, thereby reducing bacterial recognition.

Nevertheless, eliciting antibodies against bacterial colonization factors have been proposed as a vaccination strategy to prevent pathogenic *E. coli* infection (18). Antibodies against the T3SS proteins, such as EspA, EspB, and EspD, have been detected in the serum from patients with diarrheagenic *E. coli* infections, demonstrating their immunogenic potential (19–22). Previous studies have shown EspB as a target for vaccine formulations in the veterinary field, ranging from transferred maternal colostral antibodies and intramuscular immunization in cattle (18, 23), to oral and intranasal immunization in mice (24, 25). Vaccine development against enteric pathogens that are able to induce strong mucosal immune responses capable of preventing intestinal colonization are of great importance to protect humans and animals from pathologies (21, 23).

Herein, we synthetically constructed a hybrid recombinant EspB (rEspB), representative of all known variants to date, and characterized its secondary structure, which allowed us to propose an immunogenic domain.

## Materials and Methods

### Bacterial Strains, Plasmid, and Supplies

The *E. coli* strains used were DH5α [F^−^Φ80*lac*ZΔM15 Δ(*lac*ZYA-*arg*F) U169 *rec*A1 *end*A1 *hsd*R17 $\left({\text{r}}_{\text{k}}^{-},{\text{m}}_{\text{k}}^{+}\right)$ *pho*A *sup*E44 *thi*-1 *gyr*A96 *rel*A1 λ^−^] and BL21 (DE3) [F^−^*ompT hsdS*_B_ $\left({\text{r}}_{\text{B}}^{-},{\text{m}}_{\text{B}}^{-}\right)$ *gal dcm* (DE3)] from Invitrogen (CA, USA). The plasmid used was pET28a(+) containing a 6-histidine tag (His-tag) at both the N- and C-terminal from Novagen (Darmstadt, Germany). T4 ligase and T4 buffer DNA ligase (2×) were purchased from Promega Corporation (WI, USA). The enzymes used (*Bam*HI and *Hin*dIII) and the induction agent isopropyl β-d-1-thiogalactopyranoside (IPTG) were obtained from Thermo Scientific (MA, USA). The monoclonal anti-polyHistidine antibody produced in mouse, anti-mouse IgG (whole molecule) peroxidase antibody and 3′3′-diaminobenzidine (DAB) were purchased from Sigma-Aldrich (MO, USA). Luria–Bertani (LB) medium was from BD (NJ, USA), and kanamycin from Gibco (MA, USA).

### Synthetic Gene Design

**Figure 1:**
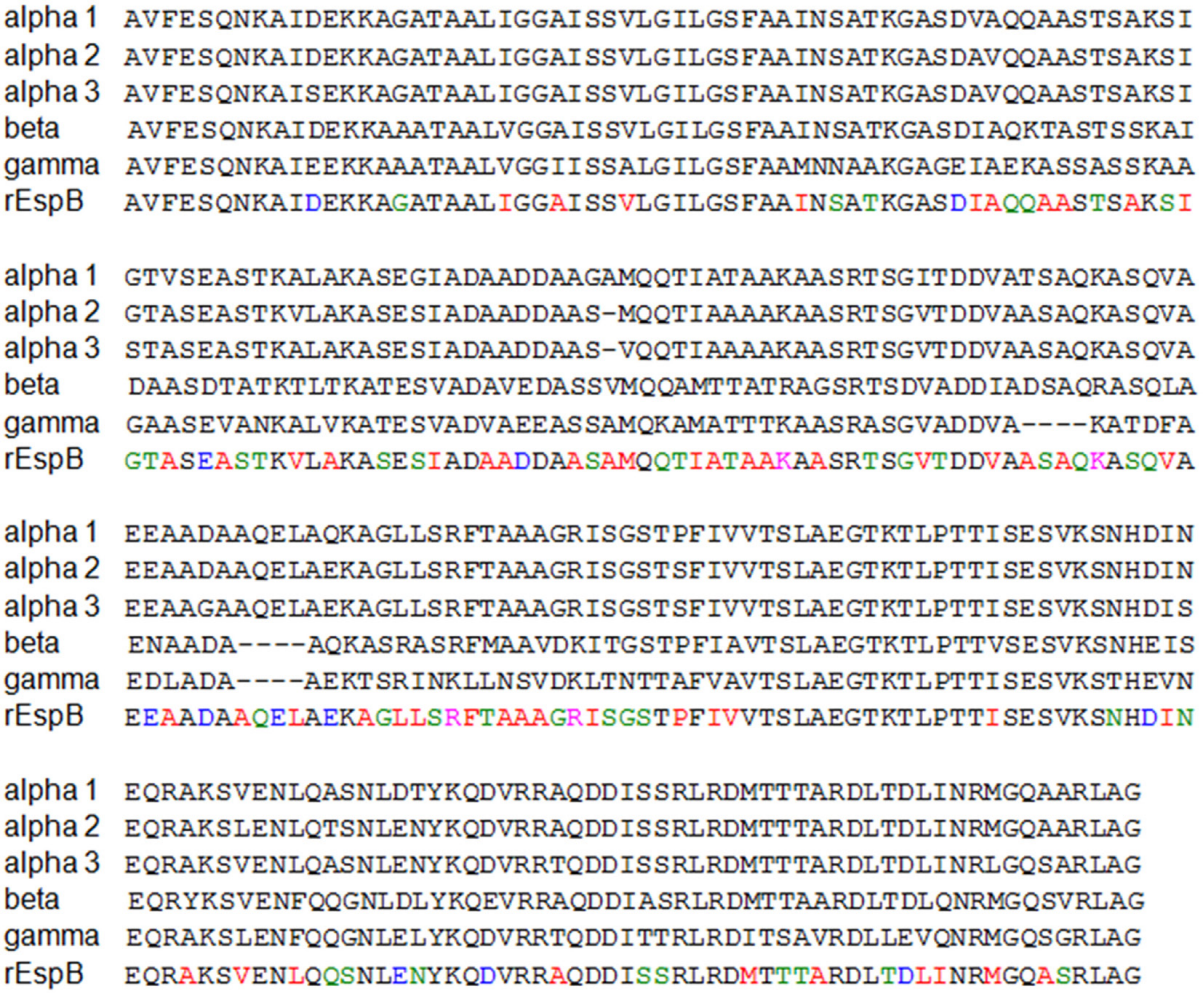
**Recombinant *Escherichia coli*-secreted protein B (rEspB) conserved domain**. Alignment of all EspB variants known to date. Shown in black are the conserved regions and in colors the divergent amino acids along the whole protein sequence, in which we used the most prevalent amino acids among the variants.

The EspB synthetic gene was developed considering common regions of all known EspB variants to date, by alignment of α1, α2, α3, β, and γ EspB sequences (GenBank number: AAC38396.1, AEW69664.1, AEW69663.1, CAA74174.1, and CAA65654.1) using the Basic Local Alignment Search Tool (BLAST). The synthetic gene for the hybrid rEspB protein was assembled on the basis of the most prevalent amino acids among the variants for each position of the protein sequence (Figure 1). The restriction enzymes were selected with the support program BioEdit version 7.2.0, and the restriction sites for the enzymes *Bam*HI and *Hin*dIII were inserted into the conserved sequence upstream and downstream, respectively (Figure S1 in Supplementary Material), while no stop codon was added in the sequence. The predicted recombinant protein had a molecular weight of 24.6 kDa. The EspB gene was manufactured by GenScript (NJ, USA) and cloned into pUC57 vector.

### Cloning

Chemically competent *E. coli* BL21 (DE3) were obtained using the Chung and Miller protocol, with modifications (26). The gene of interest was excised from pUC57 by restriction enzyme digestion and then cloned into the pET28a expression vector. The reaction mixture consisting of 2 µL of deionized water, 5 µL of the gene, 1 µL of the pET28a vector, 1 µL of T4 DNA ligase (3 IU), and 2 µL of T4 buffer DNA ligase (2×) was incubated at 24°C for 1 h, followed by a 4°C incubation for 18 h.

For *E. coli* BL21 (DE3) transformation, 1 µL of plasmid was incubated with 2 µL of 5× KCM buffer (0.5 M KCl, 0.15 M CaCl_2_, and 0.25 M MgCl_2_) and 7 µL of deionized water on ice for 5 min, followed by the addition of 10 µL of chemically competent cells; after 20 min, the solution was transferred to 24°C for 10 min. Subsequently, 200 µL of LB culture medium were added and the sample was incubated at 37°C for 1 h. The cells were then streaked on a LB agar plate containing 50 µg/mL of kanamycin and incubated at 37°C for 18 h.

### Expression and Purification

BL21 His-EspB transformant was cultivated in 10 mL of LB medium containing 50 µg/mL kanamycin at 37°C for 18 h with stirring at 250 rpm. The culture was then added to 500 mL of LB medium supplemented with 0.2% glucose and 50 µg/mL of kanamycin, and further grown at 37°C for 2 h at 250 rpm. After reaching an optical density of 0.6–0.8 (OD_600_), IPTG was added to a final concentration of 1 mM, and the culture was then incubated at 37°C for 4 h at 250 rpm. The cells were separated in a 5804 R centrifuge (Eppendorf, Hamburg, Germany) at 10,000 × *g* for 10 min, and the supernatant discarded. The pellet was resuspended in 60 mL of ligation buffer (20 mM Tris–HCl, pH 7.9, containing 0.5 M NaCl) with 1% 100× protease inhibitor cocktail and 50 µg/mL lysozyme, and allowed to stand in an ice bath for 30 min. The cells were lysed by three cycles of sonication for 10 min, with the amplitude set at 30% (Sonopuls Bandelin, Berlin, Germany). The lysate was centrifuged at 10,000 × *g* for 10 min and the resulting pellet was solubilized with 30 mL of buffer with 8 M urea, with stirring at 4°C for 18 h.

Purification was performed by metal affinity chromatography by gravity flow. Following urea treatment, 2 mL of Ni-NTA Agarose (Qiagen, NW, Germany) were added to the solubilized pellet and the suspension incubated at 4°C for 18 h with gentle shaking. The suspension was centrifuged at 30 × *g* for 1 min and pellet-containing agarose was gently transferred to a polypropylene column (Qiagen, NW, Germany). rEspB protein was eluted with buffer containing different concentrations of imidazole: 10, 20, 50, 100, 150, 200, 300, 400, and 500 mM. The eluted protein was refolded by long-term dialysis and subsequently concentrated by osmosis with PEG 4000. SDS-PAGE (12%) and immunoblotting were used to confirm the purification. The recombinant protein was quantified with a NanoDrop Lite Spectrophotometer (Thermo Scientific, MA, USA) and stored at 4°C. Identification of rEspB protein was performed by liquid chromatography coupled to mass spectrometry (LC–MS/MS). After SDS-PAGE analysis, protein bands were subjected to *in gel* trypsin digestion (27) and the resulting peptide mixture was analyzed by LC–MS/MS as described elsewhere (28). LTQ-Orbitrap Velos raw data were searched against a target database (UniProt restricted to *E. coli*; 22,940 sequences) using Mascot search engine (Matrix Science, UK).

### Structure Analysis

Protein secondary structure was confirmed after refolding by circular dichroism (CD). The CD spectra were recorded between 190 and 260 nm using a quartz cuvette (0.1-mm path length) in a JASCO J-810 Spectropolarimeter (Jasco Corporation, Japan). After buffer-background subtraction (10 mM sodium phosphate buffer, pH 8.0), the CD data were converted to mean residue ellipticity [θ] units (degree × cm^2^ dmol^−1^). CD spectra were obtained at three different pH (7.0, 8.0, and 9.0) and at temperatures ranging from 5 to 95°C. The results were analyzed on the online server DICHROWEB (29–31), using the analysis program CDSSTR (32–34).

### Epitope Mapping

Antibody-binding epitopes were determined by designing a CelluSpots^®^ peptide array (INTAVIS Bioanalytical Instruments AG, NW, Germany), with 384 dots containing the full protein sequence divided in peptides with 11 amino acids/dots, having 8 overlapping amino acids. The sequences in the array were derived from the rEspB protein, which represents the α variants, as well as the full β and γ sequences (Table S1 in Supplementary Material) (5, 10, 11, 13). The assay was performed following the manufacturer’s recommendations: briefly, the slides were blocked by immersion in 1% BSA at 4°C for 18 h with shaking. Anti-EspB monoclonal antibody (mAb) (10 µg/mL) and polyclonal antibody (pAb) (30 µg/mL) were incubated at 24°C for 4 h with stirring. The slides were washed three times with 0.05% Tween in PBS (0.01 M, pH 7.2) for 5 min. The slides were then incubated with peroxidase-conjugated anti-mouse IgG antibody (1:5,000) at 24°C for 2 h with stirring. Detection was performed with DAB and hydrogen peroxide and the reaction stopped with distilled water.

### Antibodies

Polyclonal serum was obtained from a New Zealand White female rabbit (60 days old) after immunizing intramuscularly, three times with 2-week intervals, using a dose of 100 µg of rEspB protein adsorbed to 2.5 mg alum (Al^3+^) as adjuvant. Serum was obtained 45 days after the first immunization. Immune serum reactivity was tested by indirect ELISA (35). Serum samples were obtained just before immunization from the auricular vein, which were used as the negative control in specific antibody evaluation. The anti-EspB mAb A5 was raised in the present study as in previous work by our group where mAb 4D9 was obtained (17).

## Results

### rEspB Protein

**Figure 2:**
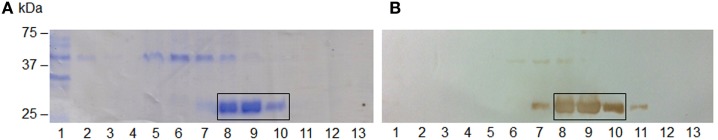
***Escherichia coli*-secreted protein B (EspB) protein SDS-PAGE and immunoblotting purification profile**. **(A)** 12% SDS-PAGE; **(B)** immunoblotting; (1) flow through; (2–4) wash with 20 mM Tris–HCl, pH 7.9, containing 0.5 M NaCl; (5–13) elution fractions with increasing imidazole concentration, respectively, 10, 20, 50, 100, 150, 200, 250, 300, and 400 mM. The steps where EspB was effectively eluted are highlighted.

The hybrid rEspB protein was obtained from the *E. coli* BL21 transformed with a plasmid harboring the hybrid *espB* gene. Restriction analysis confirmed that all clones had the same plasmid profile, and synthetic gene cloning was confirmed by sequencing. The protein was expressed in inclusion bodies; thus, urea treatment was necessary before the purification process. Since there was no stop codon in the cloned gene and since pET28a was the expression vector, the recombinant protein was expressed with two His-tag tails, one at each end of it. The protein was eluted using different imidazole concentrations, with effective elution occurring between 100 and 200 mM imidazole (Figure 2).

### EspB Protein Characterization

To confirm the identity of rEspB, protein bands indicated in Figure 2A were subjected to mass spectrometric analysis, which resulted in the identification of nine tryptic peptides (^95^AGATAALIGGAISSVLGILGSFAAINSATK^124^, ^216^AGLLSR^221^, ^222^FTAAAGR^228^, ^229^ISGSTPFIVVTSLAEGTK^246^, ^247^TLPTTISESVK^257^, ^258^SNHDINEQR^266^, ^288^AQDDISSR^295^, ^298^DMTTTAR^304^, and ^305^DLTDLINR^312^), confirming the expression and isolation of protein EaeB (UniProt entry EAEB_ECO27).

### Secondary Structure Prediction under Different Conditions

**Figure 3:**
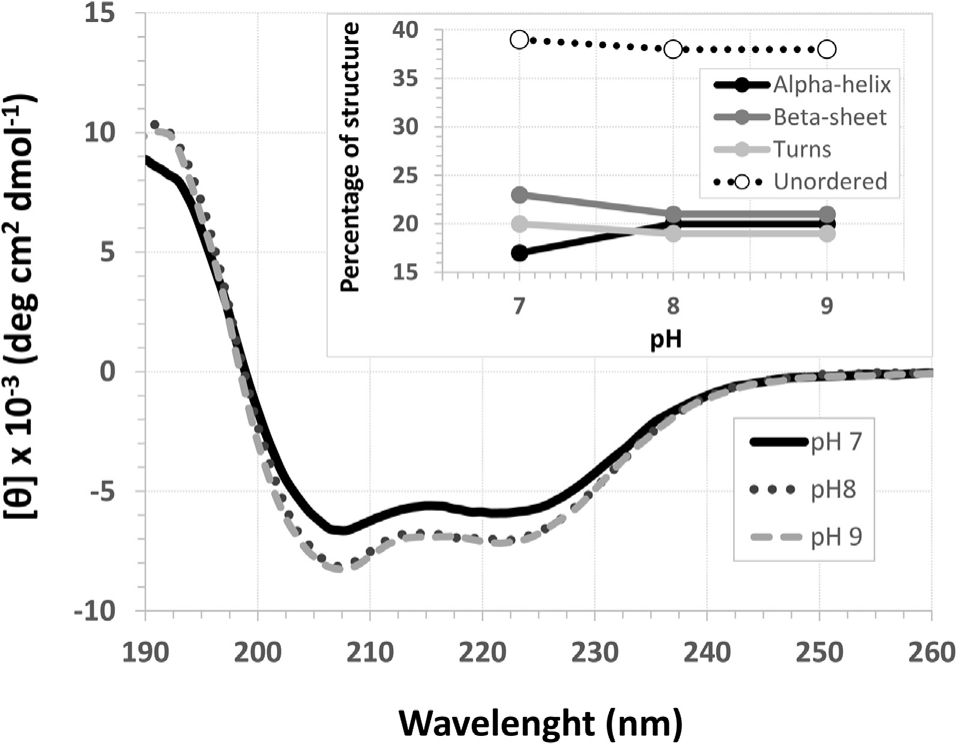
**pH circular dichroism spectra profile of *Escherichia coli*-secreted protein B (EspB) protein**. EspB profile at pH 7.0, 8.0 and 9.0, showing maintenance of structure at pH 8.0 and 9.0. Inset shows the percentage of secondary structure for each pH tested.

**Figure 4:**
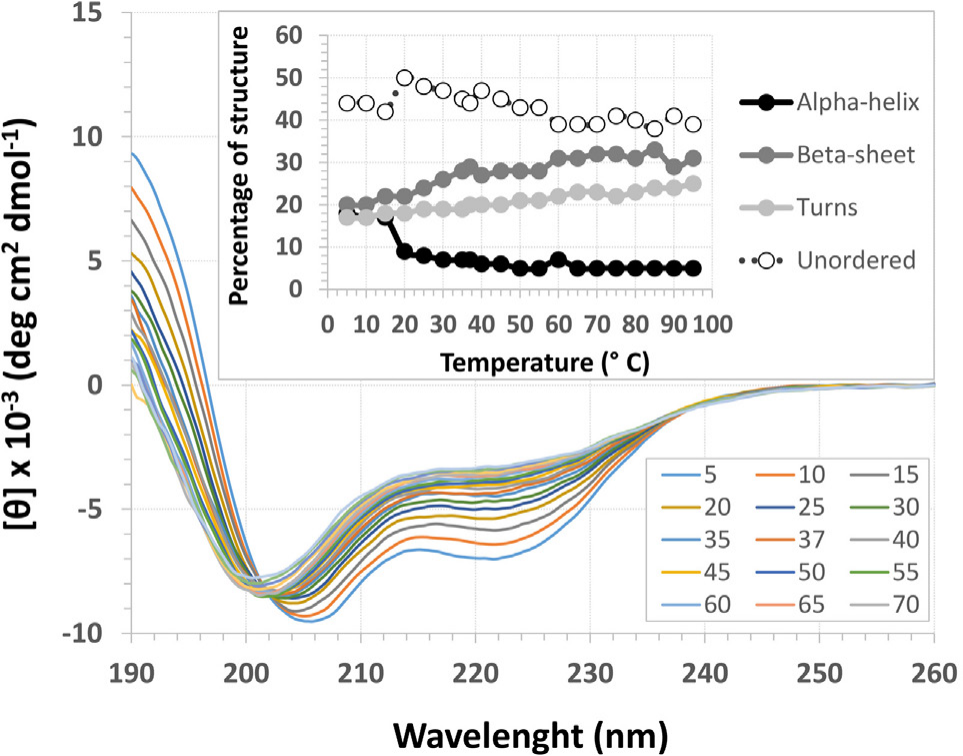
**Temperature circular dichroism spectra of *Escherichia coli*-secreted protein B (EspB) protein**. Secondary structure of EspB at different temperatures ranging from 5 to 95°C, showing the loss of structure when approaching 0 mean residue ellipticity [θ]. Inset shows the variation in the percentage of secondary structure for each temperature tested.

The EspB CD spectra showed a negative ellipticity band at 222 nm, which corresponds to α-helix structure (36). This secondary structure was observed at all pH tested; however, ellipticity was closer to 0 at pH 7.0, indicating less α-helix content under this condition when compared to pH 8.0 and 9.0. Indeed, deconvolution analysis showed a higher level of unordered content at pH 7.0 and, on the other hand, an increase in α-helix content at pH 8.0 and 9.0 (Figure 3; Table S2 in Supplementary Material). Effects on protein secondary structures were observed at a higher temperature, indicating protein denaturation by heat (Figure 4; Table S3 in Supplementary Material).

Treatment with temperatures over 60°C resulted in altered secondary structure stability, showing this to be the necessary temperature to denature the rEspB protein. There was a slight reversible loss of secondary structure after heating to 95°C and gradual cooling to 5°C. Close to the storage temperature, at 5°C, EspB protein secondary structure exhibited approximately 18% α-helix, 20% β-sheet and 20% turns, and 42% irregular structures. As the temperature increased, the proportion of turns and irregular structures tended to be the same, while the α-helix content decreased and β-sheet content increased. Above 60°C, there were no more alterations in secondary structure, with α-helix around 5% and β-sheet 31%.

### Antibody-Binding Epitopes and Probable Immunogenic Domain of EspB

The anti-EspB pAb generated against the hybrid rEspB was able to recognize several dots on the peptide array, suggesting that those epitopes are involved in antibody binding. The pAb reacted with 11 points on α EspB, 4 on β EspB, and 5 on γ EspB (Table 1). Several spots were adjacent to each other, which resulted in three sequences for the α variant (^198^ASQVAEEAADA^208^, ^231^GSTPFIVVTSLAEG^244^, and ^264^EQRAKSVENLQASNLDTYKQDVRRAQDDISSR^295^) and the same two sequences for the β and γ variants (^232^STPFIVVTSLAEGT^245^ and ^271^ENLQASNLDTYKQDVRRAQDDISS^294^). The common domain between all sequences could be defined as ^232^STPFIVVTSLAEG^244^ and ^271^ENLQASNLDTYKQDVRRAQDDISS^294^.

**Table 1:** **Binding epitopes of individual antibodies recognized by peptide array**.

*Escherichia coli*-secreted protein B (EspB) variant	Polyclonal antibody (pAb)	Monoclonal antibody (mAb) 4D9	mAb A5
α EspB	ASQVAEEAADA		
	VAEEAADAAQE		
	GSTPFIVVTSL		
	PFIVVTSLAEG		AVFESQNKAID
	DINEQRAKSVE	DAAQELAEKAG	EAADAAQELAE
	EQRAKSVENLQ	ARDLTDLINRM	DAAQELAEKAG
	VENLQQSNLEN		ARDLTDLINRM
	LQQSNLENYKQ		LTDLINRMGQA
	SNLENYKQDVR		
	KQDVRRAQDDI		
	VRRAQDDISSR		
β EspB			AITASAINSSL
	TPFIAVTSLAE	KGASDIAQKTA	GKMVRILQDYQ
	NFQQGNLDLYK	MTTAARDLTDL	VRILQDYQQQQ
	QGNLDLYKQEV	AARDLTDLQNR	LQDYQQQQLSQ
	EVRRAQDDIAS		QLAVFESQNKA
			AARDLTDLQNR
γ EspB	TTAFVAVTSLA		
	FVAVTSLAEGT	AAGAASEVANK	
	ENFQQGNLELY	AASEVANKALV	AAGAASEVANK
	LYKQDVRRTQD		
	QDVRRTQDDIT		

The mAbs 4D9 and A5 were used for comparison with the pAb, since they were obtained against the α EspB variant. Individually, mAb 4D9 reacted with 2 points on α EspB, 3 on β EspB, and 2 on γ EspB, and mAb A5 reacted with 5 points on α EspB, 6 on β EspB, and 1 on γ EspB (Table 1). The common region between the mAbs was two dots on α EspB (^127^DAAQELAEKAG^137^ and ^223^ARDLTDLINRM^233^), one dot on β EspB (^295^AARDLTDLQNR^305^), and one dot on γ EspB (^139^AAGAASEVANK^149^) (Figure S2 in Supplementary Material).

Another BLAST alignment of the two major sequences recognized by the pAb was performed against non-redundant protein sequences (nr) within bacteria (taxid:2) to evaluate if the sequences actually correlated with EspB and had 100% identity to the enterobacterial EspB protein from *E. coli* (data not shown).

## Discussion

*Escherichia coli*-secreted protein B protein is translocated into the host cell through a T3SS and together with EspD is responsible for assembling a multimeric pore in the eukaryotic membrane, contributing to the hallmarks of the A/E lesion. Due to this characteristic, EspB has a major importance in bacterial virulence and its detection can be used as a diagnostic tool for diarrheagenic *E. coli* infections. However, the allele diversity of EspB in immune response leads to specific antibodies that may not be able to recognize different variant bacterial strains.

Considering that EspB can be found in EPEC and EHEC, both related to severe diarrhea cases in human, its diagnosis and prevention are of great value for public health. Besides, since cattle are a natural reservoir of EHEC and a source for human infection, veterinary diagnosis and prevention are of major importance as well (37, 38). Vaccine strategies to prevent EPEC and EHEC infections employing EspB as an antigen have been proposed in mice and cattle, contemplating the veterinary field (18, 23–25). We obtained a hyperimmune serum from rabbits recognizing EspB, demonstrating the protein antigenic ability. Furthermore, it is known that in cases of infection, antibodies against EspB can be found in human serum (19–21), thus making EspB a target protein for the development of diagnostic tests and vaccine formulations.

In addition, EPEC and EHEC EspB protein showed less than 50% identity when compared to the homologs from *Salmonella enterica, Yersinia enterocolitica*, and *Pseudomonas aeruginosa*, suggesting that a diagnostic test for EspB can be specific for EPEC and EHEC. Based on EspB detection, previous studies proposed diagnostic methods for human infection by EPEC and EHEC, such as an immunochromatographic test (16) and latex agglutination test (15, 17), while ELISA was proposed for herd diagnosis (37). All of those methods rely on antibody recognition ability, and they used heterologous EspB protein obtained from the *espB* gene amplified from specific strains, which can result in ineffective antigen recognition, since other EspB variants may not be effectively recognized by the antibody. It is worth mentioning that mAb 4D9, described by our group, reacted with 2 points on α EspB, 3 on β EspB, and 2 on γ EspB, thus supporting our previous results, which were 97% sensitivity, 98% specificity, and 97% efficiency for a rapid agglutination latex test. All EspB variants were detected by the mAb in the peptide array assay; however, when used for strain recognition, the mAb did not recognize the different variants (17). One hypothesis is that in the peptide array the epitopes are linearized; thus, the antibody identifies small parts of an epitope that is recognized when the protein presents quaternary structure.

Therefore, the use of an EspB protein that comprises and represents all known variants as an antigen and for antibody development continues to be necessary. Thus, herein, a hybrid recombinant protein EspB was developed and characterized in terms of secondary structure, thermostability, and immunogenic region.

For that purpose, a rEspB protein comprising all known variants was designed and expressed. The His-tag tails did not affect secondary structure. The percentage of unordered structures decreased as pH increased from 7.0 to 8.0 and then remained stable when pH increased from 8.0 to 9.0. This finding suggests that the secondary structure of EspB has greater proportions of α-helix and β-sheet and turns at pH 8.0 and 9.0 when compared to pH 7.0 (Figure 3; Table S2 in Supplementary Material). Moreover, our data suggest that this increase in the percentage of ordered secondary structures was due to an increase in α-helix content; on the other hand, the percentage of β-sheets and turns was slightly reduced when pH increased. This increase in the proportion of ordered secondary structures at pH 8.0 and 9.0 may produce a change in the biological activity (in efficacy or even specificity) of this protein at this basic pH. EspB is mostly composed of irregular structures, followed by almost equal proportions of α-helix and β-sheet and turns. In regard to temperature stability, the α-helix content tended to decrease with temperature increase, while the β-sheet proportion increased with temperature (Figure 4; Table S3 in Supplementary Material). Either way, at temperatures above 60°C, the change in secondary structure halts, leading us to believe that, in this range, EspB is heat denatured, showing a heat-sensitivity characteristic.

Furthermore, the epitope mapping assay analyses allowed not only the characterization of antibody/epitope binding described here but also the proposal of an EspB immunogenic consensus domain. pAb is generated by humoral immune response, and their recognizing domain shows the EspB protein sites capable of activating the immune system. We identified two common epitopes between all known EspB subtypes (^232^STPFIVVTSLAEG^244^ and ^271^ENLQASNLDTYKQDVRRAQDDISS^294^). The peptides were aligned with the EspB protein of the prototype EPEC strain E2348/69 and showed 100% identity with two regions at the C-terminus, indicating the antibody molecule binding site to EspB. These regions were also present among the binding sites of all antibodies tested, even the mAb obtained against only α EspB, indicating that they are, indeed, conserved immunogenic domains for this protein.

This recombinant protein can be used in clinical applications, such as antigen for antibody production, enabling not only the diagnosis of A/E-producing pathotypes by EspB protein recognition but also as an alternative therapy for the disease by eliciting neutralizing antibodies against different EspB variants. Moreover, rEspB itself can serve as an antigen in a vaccine formulation to generate host antibodies able to prevent disease occurrence. In conclusion, we developed and obtained a hybrid rEspB protein capable of inducing antibody response against all known EspB subtypes, which can be a promising tool to be used as antigen for antibody development for the diagnosis and prevention of A/E lesion-producing pathogens.

## Ethics Statement

The experiments were conducted in agreement with the Ethical Principles in Animal Research, adopted by the Brazilian College of Animal Experimentation, and they were approved by the Ethical Committee for Animal Research of Butantan Institute (5492021015).

## Author Contributions

RP conceived and designed the experiments, analyzed the data, contributed reagents/materials/analysis tools, and wrote the paper. BC conceived and designed the experiments, performed the experiments, analyzed the data, and wrote the paper. LR and DL conceived and designed the experiments, analyzed the data, and wrote the paper. EC performed the experiments, analyzed the data, and wrote the paper.

## Conflict of Interest Statement

The authors declare that the research was conducted in the absence of any commercial or financial relationships that could be construed as a potential conflict of interest.

## Supplementary Material

Figure S1 **Sequence coding for *Escherichia coli*-secreted protein B (EspB) protein**. The sequence has 245 amino acids, and 241 encoding for EspB protein. The underlined sequences represent the cleavage sites of the restriction enzymes *Bam*HI, upstream, and *Hin*dIII, downstream.

Figure S2 **Antibodies binding epitope by peptide array**. The dots indicate the linear sequence of 11 peptides that the monoclonal antibody (mAb) 4D9, mAb A5, and polyclonal antibody (pAb) bind, respectively. From top to bottom, the array was designed containing the recombinant *Escherichia coli*-secreted protein B (rEspB) sequence, which represents the α variants and β and γ sequences, highlighted in bars. The two sides of the slide are duplicate of the sequence.
