# Supplementary figures and images for: Immunogenic Domains and Secondary Structure of Escherichia coli Recombinant Secreted Protein Escherichia coli-Secreted Protein B
Source: Front Immunol. 2017 Apr 24;8:477. doi: 10.3389/fimmu.2017.00477 (PMC5402224; doi:10.3389/fimmu.2017.00477)

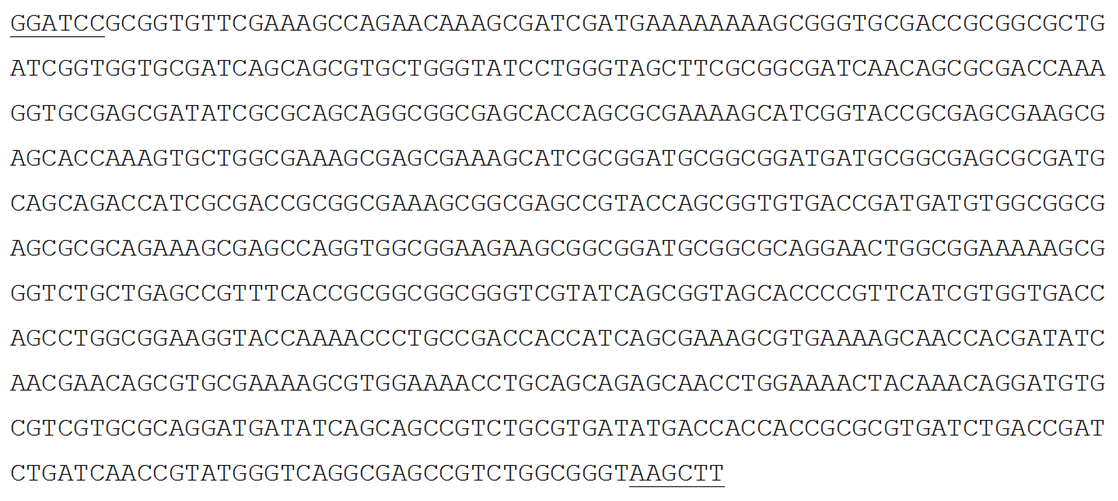

Supplement: Figure S1 — Sequence coding for Escherichia coli-secreted protein B (EspB) protein. The sequence has 245 amino acids, and 241 encoding for EspB protein. The underlined sequences represent the cleavage sites of the restriction enzymes BamHI, upstream, and HindIII, downstream. [file Image_1.TIFF]

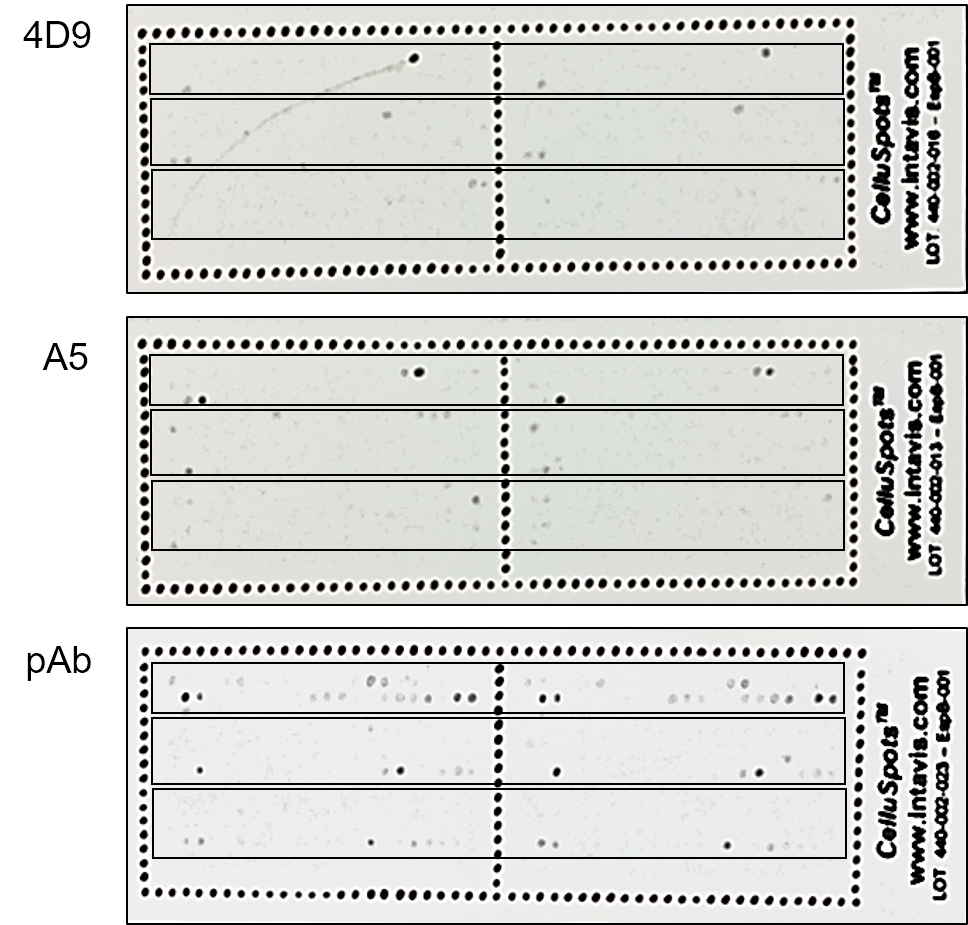

Supplement: Figure S2 — Antibodies binding epitope by peptide array. The dots indicate the linear sequence of 11 peptides that the monoclonal antibody (mAb) 4D9, mAb A5, and polyclonal antibody (pAb) bind, respectively. From top to bottom, the array was designed containing the recombinant Escherichia coli-secreted protein B (rEspB) sequence, which represents the α variants and β and γ sequences, highlighted in bars. The two sides of the slide are duplicate of the sequence. [file Image_2.TIFF]
